# Quantifying Clinically Meaningful Point-of-Care Ultrasound Interpretation Discrepancies Using an Emergency Department Quality Assurance Program

**DOI:** 10.7759/cureus.42721

**Published:** 2023-07-31

**Authors:** Steven Skitch, Dean Vlahaki, Andrew Healey

**Affiliations:** 1 Department of Medicine, Division of Critical Care Medicine, McMaster University, Hamilton, CAN; 2 Department of Medicine, Division of Emergency Medicine, McMaster University, Hamilton, CAN

**Keywords:** quality improvement, image archiving, quality assurance, point of care ultrasound, emergency medicine

## Abstract

Objectives: Emergency medicine professional associations recommend that quality assurance (QA) programs be implemented wherever emergency department (ED) point-of-care ultrasound (POCUS) is in use. The purpose of this study is to identify the rate of clinically meaningful interpretation discrepancies between initial ED POCUS interpretation and a gold standard using a QA program in a Canadian academic ED.

Methods: All POCUS examinations completed in our ED are subject to a QA process. The results of all POCUS examinations undergoing this process from July 1, 2014, to June 30, 2015, were retrospectively reviewed. Four blinded abstractors collected data with a standardized tool after a training session. Information regarding patient demographics, POCUS indication, emergency physician initial POCUS interpretation, physician POCUS expertise, the presence of an interpretation discrepancy, and whether the discrepancy was clinically meaningful was abstracted. The proportion of interpretation discrepancies, clinically meaningful discrepancies, discrepancies requiring remedial action, and differences in discrepancy rates between non-expert and expert sonographers were analyzed.

Results:A total of 2,869 POCUS studies were included for review, with 2,668 in the final data set after exclusions. In total, only 1.4% of all scans contained an interpretation discrepancy. The rate of clinically meaningful discrepancies was 0.5%, and the rate of scans requiring remedial action was 0.1%. Overall, 85.5% of all scans were performed by four POCUS expert physicians, with the remainder by a non-expert. Scans performed by non-expert sonographers were significantly more prone to discrepancies than those performed by experts. No single scan indication was more prone to discrepancy.

Conclusions: The overall ED POCUS interpretation discrepancy rate and clinically meaningful discrepancy rate identified using our QA process were very low. The findings are limited by the small group of expert sonographers completing most scans.

## Introduction

Point-of-care ultrasound (POCUS) is considered an essential tool in the delivery of excellent emergency department (ED) care. Moreover, the establishment of robust imaging documentation and quality assurance (QA) processes is viewed as fundamental to ED POCUS programs [1-3]. Myriad advantages of image archiving have been listed, including enrichment of POCUS education, enhancement of image quality and interpretation, as well as fostering improved communication among healthcare providers. While research has been conducted on the rate of imaging misinterpretation among plain radiograph ED QA programs [4-5], there remains an area of opportunity in assessing the impact of a QA process specifically tailored to POCUS.

The purpose of this study is to evaluate the outcomes of a POCUS QA process in an academic ED. Specifically, quantifying the rate of clinically meaningful POCUS interpretation discrepancies discovered through a QA process is the primary outcome. Secondary objectives include determining the overall discrepancy rate, the difference in discrepancy rate between non-expert and expert sonographers, and the discrepancy rate for common POCUS indications. The results of this study may inform the establishment of POCUS QA programs in other jurisdictions or the refinement of existing programs and identify individual scan indications that are particularly prone to misinterpretation.

## Materials and methods

Study design and setting

The St. Joseph’s Health Care Hamilton ED is an urban academic hospital in Hamilton, Ontario, Canada. The emergency physicians that staff this ED host a POCUS subspecialty training program (hereafter referred to as a POCUS fellowship) in addition to both a Royal College of Physicians and Canadian College of Family Physicians EM residency training program.

All POCUS examinations completed in the St. Joseph’s ED are archived using the program Q-Path (Q-Path version 5.5.458; Telexy Healthcare). A corresponding POCUS interpretation is saved in the patient’s medical chart and Q-Path at the time of image interpretation. None of the scanning physicians were aware of the intention to complete this study at the time of scanning.

For the purposes of this study, we considered an expert sonographer to be an emergency physician who holds American Registry for Diagnostic Medical Sonography (RDMS) credentials. All other emergency physicians were considered non-experts. In our ED, the acquisition of images for a significant proportion of the scans is carried out by the POCUS fellows. Exams completed by the POCUS fellows are reviewed with a staff physician shortly after image capture, either in person or remotely via Q-Path using an on-call system. When POCUS fellows perform scans, the attending physician signs off on the final imaging interpretation recorded in Q-path. Only clinically indicated scans are permanently stored in Q-Path and all educational scans are removed after review.

All POCUS examinations stored in Q-Path are subject to a QA process. The QA process is overseen by the POCUS program director (AH) but executed by the POCUS fellows (SS, DV). Examinations are assessed for technical adequacy, and the emergency physician's interpretation of each scan is compared against the best available gold standard from a review of the patient’s chart to identify discrepancies. The selection of the best available gold standard followed a predetermined hierarchy. Priority for gold standard selection is first given to pathology specimens (e.g., appendix at laparoscopy), then patient clinical course (e.g., discharge diagnosis), followed by radiology-performed sonography, other radiology-performed imaging modality, and finally, QA overread by the POCUS program director when no more objective comparator is available, in this order.

The examination is classified as true positive, true negative, false positive, false negative, technically limited, or not classifiable (missing data). No reviewer performs the QA process on their own POCUS examinations when an objective gold standard is unavailable and therefore requires QA overreading. When discrepancies are identified, the POCUS director evaluates the case and determines its clinical impact. An error is classified as clinically meaningful if it altered or should have altered the patient’s clinical course, management, or follow-up. This methodology is similar to QA processes used for other imaging modalities [4-6]. A study is defined as requiring remedial action if the patient required contact by the reviewer to arrange clinical follow-up, radiology performed imaging, or there was a meaningful alteration in the patient’s management plan after the error was identified.

Data abstraction and measurements

The study was approved by the Hamilton Integrated Research Ethics Board prior to commencement (approval number 0716-c). Published recommendations for completing medical record review studies were followed, and pertinent components of these methodologies are addressed below [7-9].

All POCUS examinations and corresponding QA process results completed in the St. Joseph’s ED from July 1, 2014 to June 30, 2015 were exported from the Q-Path archive for potential inclusion. The cases were divided into four equal lots, and four medical students who were blinded to the study objectives reviewed one lot each using a standardized data abstraction tool. Prior to commencing data collection, the abstractors completed a training session and ten trial case abstractions that were reviewed with the study authors (SS, DV) to ensure a standardized process. Relevant variables were then obtained from the patient chart for each POCUS examination. Patient demographic variables, including age and sex, were obtained. In addition, a comprehensive list of St. Joseph's ED physicians was furnished to the abstractors, including their names and respective POCUS credentials. This information was utilized to determine whether the scanner could be classified as an expert or non-expert.

Clinical data, including the type of POCUS, physician initial interpretation, gold standard used for QA review, outcome of QA review, and clinical impact of outcomes, were also abstracted. We characterized four indications as core POCUS scans (pericardial effusion, abdominal aortic aneurysm, Focused Assessment with Sonography in Trauma (FAST abdominal only), and presence of intrauterine pregnancy) based on precedent from POCUS continuing medical education courses offered in Canada at the time of study completion [10-11]. Any other study type was classified as advanced.

Examinations exported from Q-Path with missing patient data (e.g., hospital patient number, patient name, sonographer name) or clinical data (e.g., emergency physician initial interpretation) rendering a decision regarding the presence of a discrepancy impossible were subject to further chart review for the missing variables. If the missing data could not be found, the examination was excluded from the final dataset.

Outcomes

The primary outcome of this study was to determine the rate of clinically meaningful POCUS interpretation discrepancies identified through the St. Joseph’s ED QA process outlined above. In addition, we examined if there was a significant difference in the rate of discrepancies between non-expert and expert sonographers or between core and advanced POCUS indications.

Analysis

Analyses were performed using SPSS Statistics Version 24 (IBM, Chicago, IL). Descriptive statistics were used to characterize the study cohort and discrepancy rates. The difference in discrepancy rates between non-expert and expert sonographers was examined using a chi-square analysis. A similar analysis was conducted to examine the difference in discrepancy rates between core and advanced POCUS indications. A random sample of 10% of study records was reviewed by a second abstractor to calculate interrater agreement using the kappa statistic (k).

## Results

Characteristics of abstracted cases

**Figure 1:**
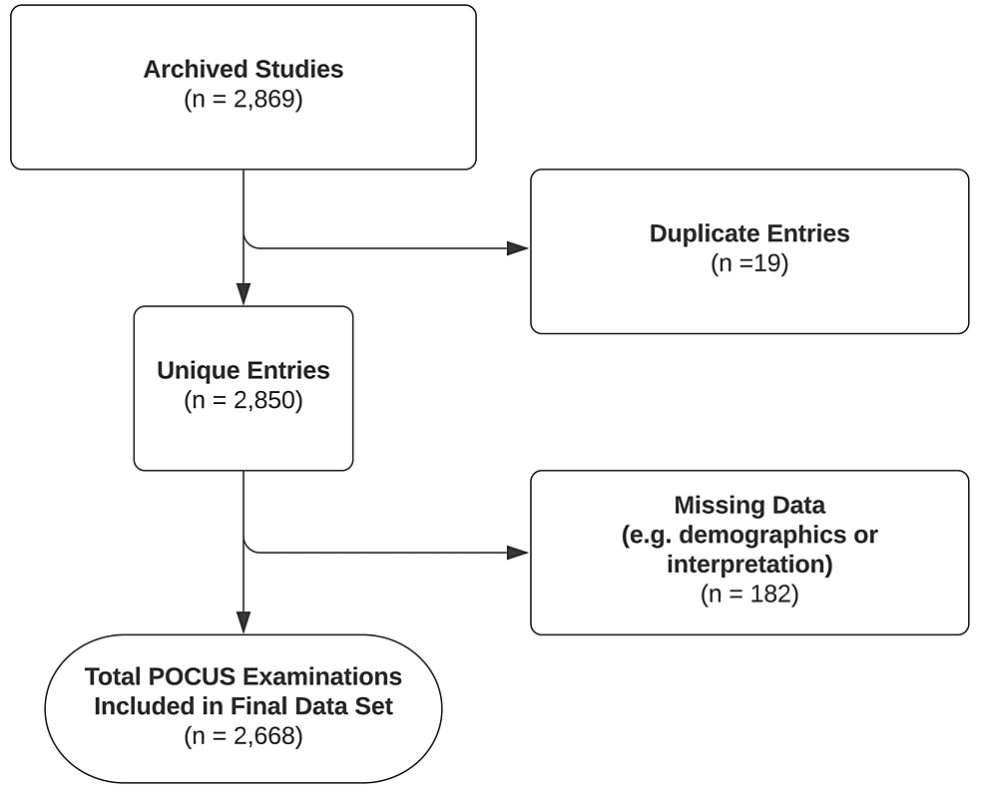
Application of exclusion criteria.

A total of 2,869 entries were identified for potential inclusion in the study period. The final dataset included 2,668 unique POCUS examinations after exclusion criteria were applied (Figure 1). The mean patient age was 50.9 years (SD 21.7), and 1,046 (39.2%) were male. There were 31 unique attending emergency physicians that completed at least one POCUS examination during the study period, and four (12.9%) of these physicians met our definition of a POCUS expert. The other 27 physicians were considered non-experts. One of these four POCUS experts was the attending physician for 2,282 (85.5%) of the POCUS examinations, and only 386 (14.5%) were completed by non-expert sonographers. The POCUS fellows were operators for 1,890 (70.8%) of the total examinations. In the current dataset, 2.2% (n = 42) of the scans completed by the POCUS fellows were reviewed by non-expert sonographers.

The frequency of scans for each POCUS indication is reported in Table 1. Core POCUS indications comprised 33% of the performed scans, while 67% of the scans were advanced indications. Non-expert sonographers were responsible for 33.2% (n = 292) of the core scans and 5.2% (n = 94) of the advanced scans. The overall POCUS test characteristics are displayed in Table 2. Notably, 2,578 (96.6%) of studies were deemed to be either true positives or true negatives, and 47 (1.8%) of studies were considered technically limited. The frequency of each gold standard used in the QA process is found in Table 3.

**Table 1:** Frequency of scans performed organized by POCUS indication. POCUS: point-of-care ultrasound.

POCUS indication		Number	Percent of total (%)
	Early pregnancy	448	16.8
	Aorta	207	7.8
	FAST	74	2.8
	Basic cardiac	148	5.5
	Advanced cardiac	458	17.2
	Skin and soft tissue	208	7.8
	Procedural	58	2.2
	Ocular	32	1.2
	Thoracic	103	3.9
	DVT	38	1.4
	MSK	43	1.6
	Renal	397	14.9
	Biliary	301	11.3
	Abdomen (other)	145	5.4
	Scrotum/testicular	4	0.1
	Other	4	0.1
	Total	2668	100

**Table 2:** Overall test characteristics for all POCUS indications (basic and advanced) as determined by the QA process. POCUS: point-of-care ultrasound.

	Number	Percent of total (%)
True positive	1138	42.7
True negative	1440	54.0
False positive	20	0.7
False negative	23	0.9
Technically limited study	47	1.8
Total	2668	100

**Table 3:** Frequency of each gold standard used in the QA process.

	Frequency (n)	Percent (%)
Clinical course or pathological specimen	306	11.5
Radiology ultrasound	701	26.3
CT scan	307	11.5
QA overread	1256	47.1
Other formal imaging	98	3.7
Total	2668	100

Main results

Discrepancies between the physician's initial interpretation and the gold standard were found in 37 (1.4%) of cases overall, including both non-expert and expert sonographers. Only 14 (0.5%) of the total studies contained a clinically meaningful error, and 3 (0.1%) of the total studies required remedial action. Of the 37 errors, 18 (48.6%) were false positives and 19 (51.4%) were false negatives. Examining the group of discrepant scans in isolation, 37.8% (n = 14) of the errors were deemed clinically meaningful, and 8.1% (n = 3) required remedial action.

When comparing core to advanced POCUS indications, there was no significant difference in the rate of discrepant scans between core (n = 11, 1.3%) and advanced (n = 26, 1.5%) POCUS indications (χ^2^(1) = 0.17, p = 0.68). However, the rate of discrepant scans completed by non-expert sonographers (n = 13, 3.4%) was significantly greater than the proportion completed by expert sonographers (n = 24, 1.1%; χ^2^(1) = 12.95, p < 0.01).

Interrater reliability was calculated for the 10% of records randomly selected for review by a second data abstractor. Interrater reliability was very good for all abstracted variables, including the presence of an error (k = 0.97), the type of gold standard used (k = 0.89), and whether an error was clinically meaningful (k = 0.80).

## Discussion

This study is the first to examine the outcomes of a POCUS QA program at an academic ED in Canada. We found that the overall discrepancy rate between an emergency physician's initial interpretation and the best available gold standard was low. Furthermore, the rate of clinically meaningful discrepancies was also low.

In comparison to the radiology literature, the rate of discrepancy identified in this study (1.4%) is similar. For example, a study of 14,046 plain radiographs obtained at two academic EDs found a discrepancy rate of 0.95%, with 0.2% deemed clinically meaningful [4]. In another pediatric emergency department study of 707 plain radiograph cases, 9.8% of radiographs were interpreted in discrepancy, with 3.1% of these being clinically meaningful [5]. These findings align with a study conducted in Australia that focused on discrepancy rates in an ED POCUS training and credentialing program. The Australian study, involving radiology auditing of specific archived studies, reported a discrepancy rate of 2.8% among the audited POCUS examinations [12].

Professional societies are consistent in highlighting the importance of POCUS image archiving and QA to improve patient care [1-2]. However, the optimal approach to image archiving and QA for POCUS in the ED remains an area of ongoing discussion and debate, with concerns about resource requirements and feasibility [13]. Similarly, practitioners cite concerns about the time burden and technical limitations as barriers to completing the recommended POCUS documentation [14]. Reducing the burden associated with image archiving and QA has been demonstrated to effectively increase POCUS utilization and documentation [15-16].

Given the finding of low clinically meaningful discrepancy rates and the labor-intensive nature of reviewing every POCUS examination as employed in the QA program used in our ED, alternate models for QA should be considered. The American College of Emergency Physicians (ACEP) has suggested reviewing 5-10% of POCUS studies, depending on the experience level of practitioners and the complexity of cases in each ED [2]. For example, QA models that review a random percentage of studies, studies that are flagged by the scanning emergency physician with diagnostic uncertainty, or only those studies completed by non-expert sonographers may be potential alternatives to auditing all POCUS examinations. Regardless of the selected approach, the guiding principles of any imaging QA process should include maintaining a non-punitive approach while revealing opportunities for quality improvement, ensuring operator competence, improving patient outcomes, allowing discrepancy trends to be identified, and having a minimal effect on physician workflow to facilitate easy participation [17]. Dependable and easily accessible image archiving is also a crucial element of a POCUS program. It not only strengthens the QA process but also allows consultants to integrate ED POCUS findings into their decision-making regarding patient management [18-19].

Limitations

While this single-center study is subject to the limitations inherent to retrospective chart reviews [7-9], other limitations warrant acknowledgment. The two POCUS fellows were operators for 70.8% of the scans, and ultimately, a group of four expert sonographers was responsible for 85.5% of the interpretations. Therefore, our results may not reflect what otherwise might be obtained in a population with a higher proportion of scans completed by non-experts. Furthermore, the definition of non-expert and expert sonographers using only RDMS credentials may underestimate the true experience of some sonographers categorized as non-experts and may decrease the non-expert discrepancy rate. Furthermore, it is important to note that we did not track the proportion of scans completed in the ED but not archived for review, commonly referred to as "phantom scans." This factor could potentially contribute to an increase in the overall discrepancy rate, especially if non-expert scanners were more prone to this phenomenon compared to experts.

In total, there were 182 scans that contained missing information, resulting in exclusion (Figure 1). If each of these examinations contained meaningful discrepancies, this would have considerably increased the primary outcome of erroneous scans identified. Also, given the low number of discrepancies overall, our study was not sufficiently powered to detect significant differences in discrepancy rates between scan types or calculate diagnostic accuracy.

The gold standard of QA overreading was used in 47% of POCUS examinations undergoing our QA process. This may limit or increase the number of discrepancies identified, depending on the reviewing physicians tendencies and biases. Finally, we chose to distinguish between basic and advanced POCUS indications based on the teaching models used in POCUS continuing medical education courses [10-11] offered in Canada at the time of study completion. This distinction may further limit the broader applicability of our results.

## Conclusions

In summary, our comprehensive QA process revealed an exceptionally low overall rate of discrepancies in POCUS interpretation, with remedial action being rarely necessary. These findings provide reassurance regarding the diagnostic accuracy of POCUS and suggest the potential feasibility of less labor-intensive approaches to QA. Future studies should focus on evaluating the clinical impact and administrative burden of alternative approaches to POCUS QA in the ED.
